# Plasma osteopontin concentrations in patients with cutaneous melanoma

**DOI:** 10.3892/or.2013.2666

**Published:** 2013-08-08

**Authors:** A. FILIA, F. ELLIOTT, T. WIND, S. FIELD, J. DAVIES, K. KUKALIZCH, J. RANDERSON-MOOR, M. HARLAND, D.T. BISHOP, R.E. BANKS, J.A. NEWTON-BISHOP

**Affiliations:** 1 Section of Epidemiology and Biostatistics, Leeds Institute of Cancer and Pathology, St. James’s University Hospital, Leeds LS9 7TF, UK; 2 Section of Oncology and Clinical Research, Leeds Institute of Cancer and Pathology, St. James’s University Hospital, Leeds LS9 7TF, UK

**Keywords:** osteopontin, ELISA, prognosis, biomarkers, melanoma

## Abstract

An effective circulating tumour marker is needed for melanoma especially with the advent of targeted therapies. Gene expression studies examining primary melanomas have shown that increased expression of osteopontin (*SPP1*) is associated with poor prognosis. Studies subsequently reported higher blood levels in melanoma patients with metastatic disease than those without. This study was designed to determine whether osteopontin plasma concentrations in disease-free patients after initial treatment predict survival. An enzyme-linked immunosorbent assay (ELISA) was used to measure osteopontin levels in stored plasma samples (N=215) from participants in the Leeds Melanoma Cohort. AJCC stage at sampling was statistically significant associated with osteopontin levels (P=0.03). Participants with untreated stage IV disease at sampling (n=10) had higher median osteopontin levels compared to those with treated stage I–III disease (n=158) (P<0.001) confirming previous findings. There was a trend for increased risk of death with increasing osteopontin levels but this was not statistically significant. If a level of 103.14 ng/ml (95th centile of healthy controls) was taken as the upper end of the normal range then 2.5% of patients with treated stage I–III (4/110), 17.6% of patients with untreated stage III (3/17) and 30% of patients with untreated stage IV disease (3/10) had higher levels. These findings suggest that plasma osteopontin levels warrant investigation as a tumour marker in a larger study in which the significance of change in levels over time should be studied in relation to detectable disease recurrence.

## Introduction

Circulating proteins have been studied as prognostic markers in melanoma (1). The prognostic value of serum lactate dehydrogenase (LDH) level in stage IV melanoma is such that it has been included in the American Joint Committee on Cancer (AJCC) staging system (2). Others have been investigated (1), particularly S100 and melanoma inhibitory antigen (MIA) but they were reported to be of no clinical utility in early-stage melanoma patients (AJCC stage I–III) (3,4).

Osteopontin, encoded by gene *SPP1*, is a multifunctional extracellular matrix glycoprotein produced by cells of many lineages, shown to be important in cancer cell adhesion, cell motility and survival (5). Osteopontin induces phosphatidylinositol 3-kinase (PI3K) activation (6,7) and acts on transcription factor nuclear factor κB (NF-κB) (8,9), potentially allowing it to regulate cell proliferation, differentiation and apoptosis.

*SPP1* gene expression was reported to be associated with melanoma progression in whole-genome gene expression profiling (10), and was later confirmed in immunohistochemical studies (11–13). Our group confirmed increased expression of *SPP1* in primary tumours to be of independent prognostic value for melanoma (14) using an agnostic approach; utilising the Illumina cDNA-mediated annealing, selection, extension and ligation (DASL) platform which measures expression of 502 cancer-related genes. Other groups reported supportive evidence using different gene expression assays (15,16) or immunohistochemistry (11,17,18).

Increased blood levels of osteopontin have been described as being associated with progression in many types of cancers (5), including cancer of the breast (19), head and neck (20) and liver (21). An increased osteopontin level was reported to be a predictor of outcome in non-small cell lung cancer (NSCLC) (22) and to be reduced after tumour resection of NSCLC (23). No association was found between osteopontin levels and disease course in mesothelioma (24). A small number of studies have shown that osteopontin levels were increased in uveal melanoma (25,26) and were highly correlated with the presence of liver metastasis (27).

Elevated osteopontin plasma concentrations have very recently been reported in two studies concerning metastatic melanoma (13,28). We report here for the first time a pilot study examining the potential prognostic utility of plasma osteopontin in early-stage disease patients (AJCC I to III) analysing the effect on risk of death from melanoma or from any cause and taking into account factors already known to be of prognostic value.

## Materials and methods

### Patients and samples

One hundred and eighty-five patients were identified from participants bled at recruitment to the Leeds Melanoma Cohort and for whom stored plasma samples were available (29). Participants were recruited to the study within 3–6 months after diagnosis, when possible. Samples were selected as follows: i) 76 samples from participants who were believed to be disease-free at venepuncture (53 treated stage I/II, 23 treated stage III), and who have not relapsed in the subsequent period of a median of 7.5 years (range, 1.1–11.2); ii) 82 from participants who were believed to be disease-free at sampling but subsequently relapsed (57 treated stage I/II, 25 treated stage III); and iii) 27 who had metastatic disease at sampling (17 untreated stage III, 10 untreated stage IV). A patient was defined as disease-free if they had had their primary melanoma excised or their lymph nodes removed and there was no known clinical evidence of further disease. A minimum period of 6 weeks between surgery and venepuncture was used based on a study in NSCLC patients which showed that osteopontin plasma levels were elevated in the period of 6 weeks after surgery possibly due to the involvement of osteopontin in wound healing (23). Thirty healthy controls were also included in the study to compare osteopontin levels with those in the normal population. No difference in age and gender was observed between controls and cases. The study was approved by the national ethics committee, MREC, and informed consent was obtained from all participants for studies on survival from melanoma.

All samples were collected into EDTA and separated by centrifugation at 1,500 × g for 15 min, prior to storage in aliquots at −80°C. Some samples stored from participants in the Leeds Melanoma Cohort had been mailed to the laboratory resulting in variation in the time from venepuncture to processing with a median of 1 day (range, 0–4 days). There are no published data for the stability of osteopontin plasma levels in stored samples. Therefore, to investigate the potential impact of delays in processing we first investigated the stability of osteopontin levels over time. In order to do this, additional plasma samples were obtained from 5 melanoma patients and 4 healthy volunteers with informed consent. Two 4-ml tubes of blood were collected from each person; one sample was processed immediately after venepuncture and plasma was stored at −80°C, and the other was processed similarly but after being left at room temperature for 4 days. In these samples osteopontin levels were measured to determine whether there was change due to variation in processing time.

### Enzyme-linked immunosorbent assay (ELISA) of plasma osteopontin

An ELISA assay kit (Quantikine; R&D Systems) was used to measure osteopontin levels according to the protocol. Prior to use the assay was validated examining intra- and inter-assay precision, parallelism and recovery, using recombinant osteopontin protein purchased from Abcam and interference as previously described (30,31). EDTA plasma samples were used for the analysis as proteolytic cleavage of osteopontin by thrombin during the clotting process occurs in serum samples. In each assay a low- and a high-quality control sample with known concentration was analysed.

### Statistical analyses

All samples were assayed in duplicate [acceptable coefficients of variation (CVs) being <10%], and the osteopontin concentrations (ng/ml) presented here are the mean of the two replicates. The potential effects of differences in sample processing time on osteopontin plasma concentrations were assessed using the results from the matched samples processed at different time-points (same day vs. 4 days after venepuncture) and analysed using the Wilcoxon matched-pairs signed-ranks test.

First, osteopontin differences between healthy controls and cases grouped according to AJCC stage at venepuncture were compared using the Kruskal-Wallis test and multiple linear regression. Second, we looked at osteopontin as a prognostic indicator for patients with no evident disease so that patients with metastatic disease at sampling were excluded from subsequent analysis.

Factors previously shown to be associated with survival in the Leeds Melanoma Cohort were assessed for association with osteopontin level: Breslow thickness, body mass index (BMI), AJCC stage, tumour site and mitotic rate, age at diagnosis, gender, tumour ulceration (ulcerated, not ulcerated), sentinel node biopsy (SNB) status, and vitamin D serum levels (nmol/l, adjusted for season) (29). Mann-Whitney U tests, Spearman correlations and Kruskal-Wallis tests were used where appropriate. Multiple linear regression was used to identify possible independent predictors of osteopontin level.

Odds ratios (OR) and 95% confidence intervals (CI) were estimated from logistic regression models for the effect of osteopontin levels on risk of death from melanoma and death from any causes. Due to the skewed frequency distribution of osteopontin, the log-transformed osteopontin level (to the base 2) was entered into the models so that the estimated OR would be interpreted as the OR associated with a doubling of osteopontin level at recruitment. Osteopontin level was also considered as a categorical variable by grouping into approximate tertiles (≤49.35, >49.35 to ≤64.34, >64.34). Both unadjusted and adjusted ORs were calculated for osteopontin; the adjustment variables were: age, gender, BMI, site of the primary, season-adjusted vitamin D level and stage at sampling. Here, the vitamin D variable was grouped into six categories, to show the effect based on 20 nmol/l increments (≤20, >20 to ≤40, >40 to ≤60, >60 to ≤80, >80 to ≤100 and >100). Secondary analyses incorporated time-to-event data and Kaplan-Meier curves were plotted. Hazard ratios (HR) and 95% CI were estimated from Cox proportional hazards models for the effect of osteopontin level on melanoma-specific survival (MSS) and overall survival (OS). An arbitrary significance level of P<0.05 was used. STATA version 10 (32) was used for statistical analyses.

## Results

### Initial validation aspects

Satisfactory validation of the ELISA assay was achieved with intra- and inter-assay precision of <10%, acceptable parallelism and recovery, and no hook effect or interference from bilirubin, haemolysis, triglycerides or rheumatoid factor (31). There was no statistically significant difference in osteopontin levels between samples processed immediately and four days later (Wilcoxon matched-pairs signed rank test; P=0.07, data not shown) with the majority of samples differing by <3% between conditions, enabling all stored samples to be used in the study.

### Cross-sectional analysis of plasma osteopontin in all cases and healthy controls

**Figure 1:**
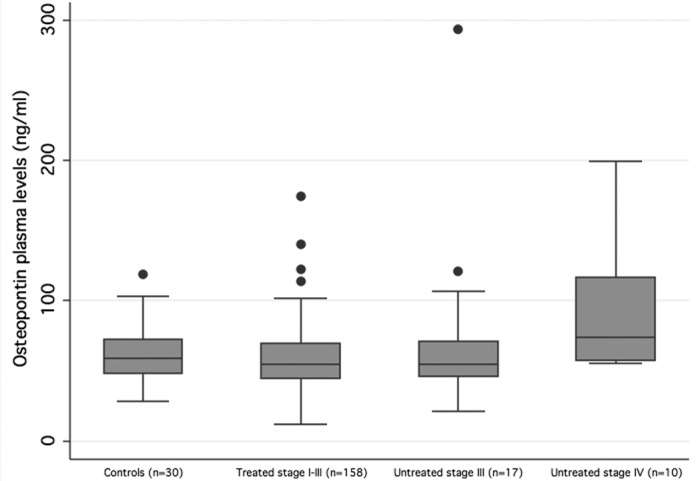
Box plots of osteopontin plasma levels in healthy controls and all cases (grouped according to AJCC stage). The edges of the box represent the 25th and 75th centiles and the whiskers represent the 5th and 95th centiles.

The normal range of osteopontin levels, as it was measured in 30 healthy controls, was 28.6–118.8 ng/ml with a median level of 59.2 ng/ml. Of the 185 melanoma patients, 158 had treated stage I–III, 17 had untreated stage III and 10 had untreated stage IV disease (median SPP1 level 54.7, 54.6 and 74.0 ng/ml, respectively) (Fig. 1). A statistically significant difference in osteopontin levels was observed between the 4 groups (Kruskal-Wallis test χ^2^=8.69; P=0.03; Fig. 1). To explore this further, we performed multiple linear regression which showed that untreated stage IV patients had significantly higher osteopontin levels than the controls and the treated stage I–III patients in age-adjusted models (P=0.004 for untreated stage IV vs. controls; P<0.001 for untreated stage IV vs. treated stage I–III patients). No significant differences were seen between controls and the treated stage I–III or the untreated stage III patient groups.

In healthy controls, 95% of samples had osteopontin levels <103.14 ng/ml. This cut-off was, therefore, taken as the upper end of normal. Patients (2.5%) with treated stage I–III (4/158), 17.6% of patients with untreated stage III (3/17) and 30% of patients with untreated stage IV disease (3/10) had levels higher than this cut-off (Fisher’s exact=0.001, data not shown). The cut-off that had been previously reported is 76 ng/ml (95th centile) (33), which is the 80th centile in our control group.

### Osteopontin in patients free of disease at sampling

Age was positively correlated with osteopontin level in the disease-free patient group (Spearman’s rho=0.2, P=0.02; Table I) and overall (Spearman’s rho=0.21, P=0.004). AJCC stage at sampling was borderline associated with osteopontin levels (P=0.06; Table I) with a higher median level noted in patients with treated stage III melanomas compared to those with treated stage I–II disease (64.3 and 54.1 ng/ml, respectively). Neither age nor stage at sampling were independent predictors of osteopontin level in a multiple linear regression model. There was no difference in osteopontin levels between SNB-positive (n=38) and SNB-negative (n=41) participants (Table I).

**Figure 2:**
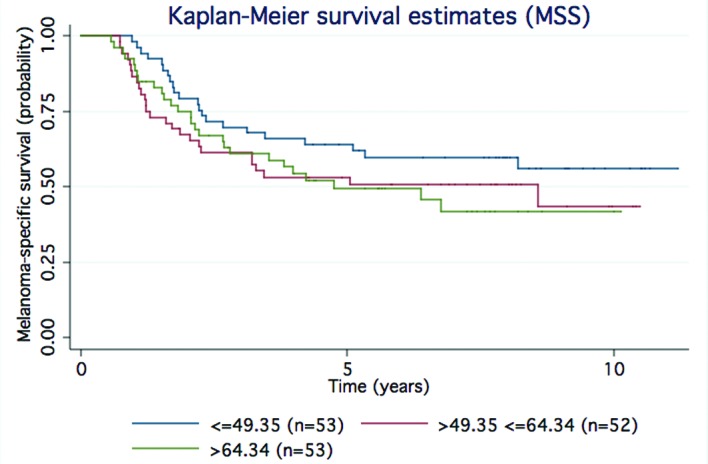
Kaplan-Meier analysis of melanoma-specific survival (MSS) estimates for osteopontin plasma concentrations in participants who were disease-free at sampling. Adjusted HR 1.26 (95% CI, 0.67–2.37), P=0.48 for middle vs. low osteopontin tertile. Adjusted HR 1.19 (95% CI, 0.62–2.28), P=0.61 for high vs. low osteopontin tertile. Adjusted models include age, gender, BMI, tumour site, season-adjusted vitamin D level and stage at sampling.

**Figure 3:**
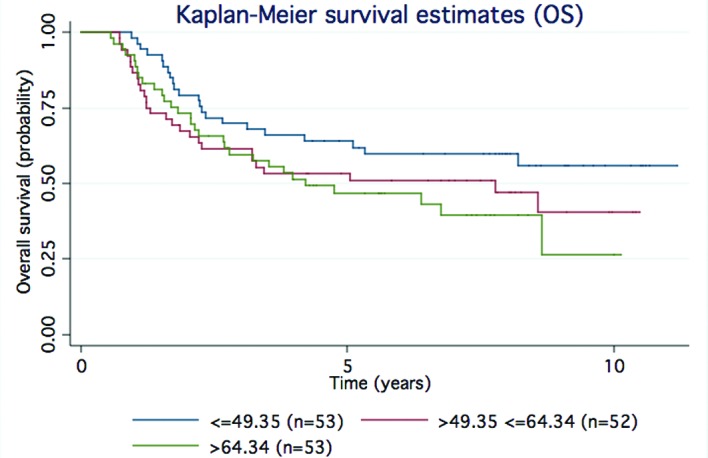
Kaplan-Meier analysis of overall survival (OS) estimates for osteopontin plasma concentrations in participants who were disease-free at sampling. Adjusted HR 1.34 (95% CI, 0.72–2.52), P=0.36 for middle vs. low osteopontin tertile. Adjusted HR 1.44 (95% CI, 0.77–2.72), P=0.26 for high vs. low osteopontin tertile. Adjusted models include age, gender, BMI, tumour site, season-adjusted vitamin D level and stage at sampling

Logistic regression showed a trend for increased risk of death from any cause with increasing osteopontin level but this was not statistically significant [adjusted OR was 1.24 (95% CI, 0.52–2.96) for the middle vs. lowest tertile, and 1.39 (95% CI, 0.56–3.42) for the highest vs. lowest tertile, adjusted for age, gender, BMI, site of primary, season-adjusted vitamin D level and stage at sampling (Table II)]. Time-to-event analyses showed support for that trend (Figs. 2 and 3 for MSS and OS, respectively). When the 95th centile cut-off was used 4/158 of the treated stage I–III patients had higher levels; 3/4 relapsed early (<1.6 years) and 1/4 after 6 years; 154/158 of the treated stage I–III patients had osteopontin levels below the cut-off and 79/154 were subsequent relapsers, 60 of which relapsed at <1.6 years.

## Discussion

We report a pilot study of osteopontin levels as a prognostic biomarker. The strength of the present study is that we looked for the first time at the prognostic value of osteopontin levels in recently diagnosed patients. The weaknesses are that this study is underpowered; only a single test sample was available, and no comparisons were made between osteopontin levels and other blood markers known to have some prognostic significance, such as LDH.

Currently, Breslow thickness, tumour ulceration, mitotic rate, lymph node metastasis, site of distant metastasis and serum LDH levels are the prognostic markers which are included in the most recent version of the AJCC staging system. Age, tumour site and gender are also powerful prognostic factors (34). Even if all these factors are considered, the variance in survival within stage is still large; thin tumours for example might progress to advanced disease (35). Therefore, there is an urgent need for identification of new prognostic biomarkers. There is also a need for a screening test to detect early recurrence now that more effective drug treatments are emerging (36,37).

A clinically useful biomarker should be measured easily, reliably and at low cost, by a sensitive and specific assay (38). A number of serological prognostic biomarkers have been studied in melanoma, but their clinical utility is still unproven. LDH serum level is most widely used (2) but is a poor marker of early recurrence and is commonly somewhat elevated in otherwise healthy individuals.

Serum levels of S100, MIA and amyloid A have been identified as potential prognostic biomarkers in advanced disease (1). However, they failed to predict outcome in early-stage disease-free disease (39) having a low sensitivity in a subgroup of patients (3).

Osteopontin plasma levels have recently been reported to be markedly increased in metastatic melanoma in two studies (13,28) and the present study provided some supportive evidence. It has been suggested that osteopontin levels might best be used in a panel of plasma markers (28). An increase in sensitivity for the detection of metastatic disease was observed when S100 plasma levels were combined with osteopontin (13).

A significant difference in osteopontin levels was observed between healthy controls and patients with melanoma grouped according to AJCC stage, but most of this difference was explained by high levels in patients with stage IV disease. Using the 95th centile measure in healthy controls as a cut-off, however, showed evidence of a trend to increased rates of results above that level with disease progression.

When only the disease-free patients were analysed in logistic regression models adjusted for known prognostic factors we saw a trend for increased risk of death with increasing osteopontin level. This provides support for the view that increased osteopontin levels might predict occult disease. Medical services in Leeds comply with the UK melanoma guidelines, which state that there is no need for routine screening (imaging) in stage I–III patients. Thus, the possibility that there may have been occult disease in our disease-free patients should be considered.

Serial measurements of osteopontin plasma levels after diagnosis may prove to be more informative than a single measurement. A study in uveal melanoma (40) showed a significant increase in osteopontin level from 12–18 months to 6–12 months prior to clinical confirmation of metastasis. When we used a measure of osteopontin level higher than 95th centile, relapse occurred in significant numbers of melanoma patients with results within the normal range. It seems unlikely, therefore, that a single measurement of osteopontin will have sufficient sensitivity and specificity for use in clinical practice. It may, however, be the case that biologically different tumours are associated with increased levels of tumour markers and that a panel of markers will be necessary to be repeated over time.

## Figures and Tables

**Table I:** Relationship between osteopontin levels and other characteristics of the participants who were disease-free at sampling (univariable analysis).

Variables	N	Median osteopontin (range)	Test statistic; P-value
Osteopontin (ng/ml)	158	54.7 (27.9–140.0)	
Age (years)	158		Spearman’s rho=0.2; 0.02
Gender	158		
Male	85	54.6 (27.9–122.4)	Mann-Whitney, z=−0.4; 0.67
Female	73	54.8 (12.0–174.4)	
BMI	155		
<18.5	1	49.5	
≥18.5 to <25	62	56.1 (28.4–174.4)	Kruskal-Wallis χ^2^=0.9; 0.82
≥25 to <30	57	53.3 (27.9–122.4)	
≥30	35	54.4 (12.0–113.9)	
Breslow thickness (mm)	156		
≤1	11	47.7 (30.8–102.2)	
>1 to ≤2	49	54.6 (31.2–174.4)	Kruskal-Wallis χ^2^=2.5; 0.47
>2 to ≤4	57	54.5 (12.0–98.4)	
>4	39	55.2 (27.9–113.9)	
Tumour site	158		
Trunk	71	53.9 (29.2–122.4)	
Head/neck	16	53.0 (29.3–102.0)	Kruskal-Wallis, χ^2^=5.7; 0.13
Limbs	55	55.2 (12.0–174.4)	
Acral/rare	16	68.0 (27.9–98.9)	
Mitotic rate (mm^−2^)	128		
<1	20	55.6 (31.2–93.0)	
1–6	69	52.4 (12.0–140.0)	Kruskal-Wallis, χ^2^=3.2; 0.20
>6	39	56.6 (35.2–98.8)	
Ulcerated tumours	158		
Not ulcerated	98	54.6 (28.4–122.4)	Mann-Whitney, z=−1.0; 0.32
Ulcerated	60	56.6 (12.0–174.4)	
Vitamin D (nmol/l)	150		Spearman’s rho=−0.1; 0.30
Stage at sampling	156		
Treated I/II	110	54.1 (27.9–122.4)	Mann-Whitney, z=−1.9; 0.06
Resected III	48	64.3 (28.4–93.2)	
SNB status	79		
Positive	38	55.2 (28.4–93.2)	Mann-Whitney, z=−0.1; 0.96
Negative	41	57.6 (30.8–122.4)	

BMI, body mass index; SNB, sentinel node biopsy.

**Table II:** Association of osteopontin plasma levels with risk of death in participants who were disease-free at sampling.

	Alive, dead from melanoma	OR (95% CI)	P-value	Alive, dead from any cause	OR (95% CI)	P-value
Continuous osteopontin						
Unadjusted model	83, 75	1.11^a^ (0.60–2.07)	0.74	79, 79	1.33^a^ (0.71–2.49)	0.37
Adjusted model	82, 65	0.85^a^ (0.42–1.70)	0.64	78, 69	1.05^a^ (0.52–2.12)	0.88
Categorical osteopontin (tertiles)						
Unadjusted model						
≤49.35	83, 75	1		79, 79	1	
>49.35 to ≤64.34		1.41 (0.65–3.04)	0.38		1.52 (0.70–3.29)	0.29
>64.34		1.46 (0.68–3.15)	0.33		1.83 (0.85–3.97)	0.12
Adjusted model						
≤49.35	82, 65	1		78, 69	1	
>49.35 to ≤64.34		1.14 (0.48–2.69)	0.77		1.24 (0.52–2.96)	0.63
>64.34		1.02 (0.41–2.52)	0.96		1.39 (0.56–3.42)	0.48

Adjusted models include age, gender, BMI, tumour site, season-adjusted vitamin D level and stage at sampling.

a OR is interpreted as the OR associated with doubling the original osteopontin variable.

## Abbreviations

SPP1: secreted phosphoprotein 1
ELISA: enzyme-linked immunosorbent assay
DASL: cDNA-mediated annealing, selection, extension and ligation
SNB: sentinel node biopsy
OR: odds ratio
HR: hazard ratio
CI: confidence interval
BMI: body mass index
LDH: lactate dehydrogenase
AJCC: American Joint Committee on Cancer
MIA: melanoma inhibitory activity
NSCLC: non-small cell lung cancer
CV: coefficient of variation
OS: overall survival
MSS: melanoma-specific survival
